# Objective Approach for Titration of Continuous Positive Airway Pressure in Patients with Obstructive Sleep Apnea Using Daytime Continuous Positive Airway Pressure Titration Based on Respiratory Movement Regularity

**DOI:** 10.3390/jcm13247603

**Published:** 2024-12-13

**Authors:** Naoto Burioka, Yuki Hirayama, Masahiro Endo, Masayoshi Oguri, Miyako Takata, Tomoyuki Ikeuchi, Akira Yamasaki

**Affiliations:** 1 Department of Respiratory Medicine, Yonago Medical Center, National Hospital Organization, Yonago 683-0006, Japan; 2 Department of Pathobiological Science and Technology, Graduate School of Medical Sciences, School of Health Science, Faculty of Medicine, Tottori University, Yonago 683-8503, Japan; 3 Division of Respiratory Medicine and Rheumatology, Department of Multidisciplinary Internal Medicine, Faculty of Medicine, Tottori University, Yonago 683-8504, Japan; 4 Endo Clinic, Izumo 693-0004, Japan; 5 Department of Medical Technology, Kagawa Prefectural University of Health Sciences, Takamatsu 761-0123, Japan

**Keywords:** apnea–hypopnea index, continuous positive airway pressure, obstructive sleep apnea, respiratory movement, titration

## Abstract

**Background/Objectives**: Continuous positive airway pressure (CPAP) is used to treat patients with obstructive sleep apnea (OSA) and has proven clinical efficacy for this condition. However, the objective method to determine the appropriate CPAP level for treatment is still unclear. Patients with OSA typically exhibit irregular respiratory efforts due to obstruction or narrowing of the upper airway during sleep. Treatment with an adequate CPAP level alleviates airway obstruction or narrowing, leading to more regular respiratory patterns. We aimed to develop an objective CPAP titration method using the respiratory regularity index (RRI) derived from respiratory movements. **Methods**: We conducted daytime CPAP titration in 10 patients with OSA. Respiratory movements were recorded by inductance plethysmography in three conditions: wake, apnea, and during CPAP therapy. The RRI of respiratory movements was calculated in each condition, and the CPAP level with the lowest RRI was identified as the appropriate air pressure level in each patient. CPAP therapy at the appropriate level determined by the study method was conducted for 2 months, after which efficacy was assessed by night polysomnography in the hospital. **Results**: The fixed air pressure level for the CPAP device was determined as 7.8 ± 0.7 cmH_2_O based on our daytime CPAP titration method using the RRI. The apnea–hypopnea index improved significantly from before CPAP therapy (43.1 ± 15.3 h^−1^) to during CPAP therapy (3.0 ± 2.1 h^−1^) (*p* < 0.006). The Epworth Sleepiness Scale score decreased significantly during CPAP therapy. The lowest percutaneous arterial oxygen saturation values in each sleep stage also improved significantly with the CPAP determined by our daytime CPAP titration method. **Conclusions**: The study findings showed that our daytime CPAP titration method based on the RRI is an effective method for determining individualized appropriate CPAP levels in patients with OSA. The RRI-based daytime CPAP titration method offers a simplified approach to determining the adequate CPAP level for patients with OSA.

## 1. Introduction

Obstructive sleep apnea (OSA) is a significant disease associated with hypertension [1], deterioration of cognitive function [2], and cardiovascular disorders [3]. Nasal continuous positive airway pressure (CPAP) is currently recognized as an effective treatment for OSA. CPAP therapy can improve OSA-induced sympathetic activation in the pathogenesis of cardiovascular disease [4]. The optimal CPAP air pressure level is determined in a hospital setting using overnight polysomnography (PSG). Traditionally, a technician or a physician subjectively determines the effective CPAP level for patients with OSA based on a full-night PSG, progressively increasing the air pressure level until apneas, hypopneas, and snoring are abolished, and percutaneous arterial oxygen saturation (SpO_2_) is normalized in all sleep stages [5]. However, this night-time manual procedure is often unavailable or insufficient in many hospitals because it is time-consuming and expensive. Several alternative approaches have been explored, including split-night diagnostic/titration studies [6,7,8], attended daytime CPAP titration with full PSG [9,10,11] and predictive equations [12], and unattended CPAP titration at home using automatic CPAP devices [13,14]. In particular, previous reports have revealed that daytime CPAP titration with full PSG is both reliable and cost-effective. On the other hand, CPAP in daytime titration is also subjectively determined by a physician when apneas, hypopneas, and snoring are abolished and SpO_2_ is normalized. Moreover, the CPAP level determined subjectively may not be the optimal pressure, and using an excessive CPAP level may lead to reduced adherence to therapy. Although an objective method for determining an optimal CPAP level is needed, few studies have investigated this issue.

Respiratory movements and efforts generally become irregular in patients with OSA [15,16], which is caused by obstruction or narrowing of the upper airway. When patients with OSA are treated with a sufficient CPAP level, the obstruction or narrowing of the upper airway resolves, resulting in more regular respiratory movements [15]. However, if the air pressure level is too high, patients using the CPAP device may become uncomfortable and wake up. In this study, we used the regularity of respiratory movements as an indicator to determine the appropriate CPAP level and developed a new objective method for daytime titrating CPAP in patients with OSA.

## 2. Methods

### 2.1. Patients

Ten male patients with OSA aged 50.9 ± 13.8 years (mean ± SD; range, 36–70 years) with a body mass index of 29.3 ± 4.8 kg/m^2^ were investigated at either Tottori University Hospital or Hitachi Memorial Hospital. All patients had undergone a night-time diagnostic PSG in the hospital prior to the study. Patients with an AHI > 15/h were included in the study. Exclusion criteria were patients with heart failure, central sleep apnea syndrome, Cheyne–Stokes respiration, major facial or pharyngeal anatomic abnormalities, previous treatment with CPAP, and previous sedative or hypnotic therapy. The Ethical Committee of Tottori University approved the protocol of this study (approval number 382), and informed written consent was obtained from all participants.

### 2.2. Measurement

#### 2.2.1. Sleep Study

A sleep study using overnight PSG was performed before the start of the study at the hospital. A PSG system (SomnoTrac Omega, SensorMedics Corporation, Yorba Linda, CA, USA) was used to record signals of electro-encephalograms (C3-A2, C4-A1), electro-oculograms, submental electro-myograms, electrocardiograms, SpO_2_ by finger pulse oximeter, snoring monitored by microphone, nasal and/or mouth flow using pressure cannula, and both chest wall and abdominal efforts. Two independent physicians each categorized the stage of consciousness on the electro-encephalogram records according to the criteria established by Rechtschaffen and Kales [17]. Apnea was defined as the cessation of airflow at the nose and/or mouth for at least 10 s, and hypopnea was defined as a >50% reduction in airflow for at least 10 s followed by a reduction in oxyhemoglobin saturation by at least 3% or an arousal event. The apnea–hypopnea index (AHI) was calculated as the sum of all apneas and hypopneas divided by total sleep time (TST) and was expressed as events per hour. Only patients with an AHI > 15/h were included in the study.

#### 2.2.2. Daytime Titration

To objectively determine the effective CPAP level, we performed daytime CPAP titration with full PSG at our laboratory from 13:00. In addition to a standard PSG, a chest band (Respiband, NonInvasive Monitoring Systems Inc., North Bay Village, FL, USA) was placed around the rib cage at the level of the nipples to measure respiratory movements by inductance plethysmography (Respisomnograph, NonInvasive Monitoring Systems Inc.). The respiratory signals obtained from the inductance plethysmograph were recorded on tape (Instrumentation tape recorder A-47, SONY, Tokyo, Japan).

First, the patient’s respiratory movements were measured while awake in the supine position, and respiratory efforts during OSA were measured with the chest band when the patient had achieved stable sleep. The patient was fitted with the nasal mask of the CPAP device (Tranquility, Respironics Inc., Murrysville, PA, USA) delivering 4 cmH_2_O pressure, after which we waited until the patient achieved stable sleep again. Sleep state was confirmed on a computer screen using signals from the PSG. The CPAP level was manually increased from 5 and 10 cmH_2_O in increments of 1 cmH_2_O at approximately 10 min intervals until apneas, hypopneas, snoring, and oxyhemoglobin desaturations disappeared. Respiratory movements were measured with the chest band at each CPAP level. If the patient clearly woke up or there was considerable air leaking from the nasal mask, this was considered to be the limit of tolerability, and the daytime titration was terminated at that point.

We used the following categories: the five sleep stages (stage I, II, III, IV, and REM) and wake [17]. The signals of respiratory effort were selected from artifact-free epochs. An artifact-free epoch of respiratory movement (3 min) was selected in each participant during the wake condition, apnea condition, and at each CPAP level in sleep stage I or II. Each epoch was digitized at a sampling rate of 10 Hz (0.1 s) with an analog-to-digital converter.

#### 2.2.3. Computation of the Respiratory Regularity Index

We developed a daytime CPAP titration method based on respiratory movements to objectively determine the appropriate CPAP device pressure in patients diagnosed with OSA in a short period of time. In general, the regularity of respiration assumes that respiratory intervals are regular and respiratory amplitudes are similar. In this method, we devised a new respiratory regularity index (RRI) measure, and calculated it from 3 min respiratory movements during wake, apnea, and CPAP levels at each 1 cmH_2_O increment when the pressure level was 5 cmH_2_O or higher. The CPAP level with the lowest RRI was identified as the appropriate pressure for treatment in this study.

The RRI was defined as follows:

RRI = log_10_ (coefficient of variation in respiratory intervals multiplied by coefficient of variation in respiratory amplitudes).

Coefficient of variation in respiratory intervals = (standard deviation of respiratory intervals from time-series data divided by mean of respiratory intervals from time-series data) multiplied by 100 (%).

Coefficient of variation in respiratory amplitudes = (standard deviation of respiratory amplitudes from time-series data divided by mean of respiratory amplitudes from time-series data) multiplied by 100 (%).

Respiratory interval was defined as the respiratory cycle time between adjacent peaks of the measured respiratory movement curve, and respiratory amplitude was defined as the magnitude from the peak to the trough point of the respiratory movement curve. We manually calculated the value of the coefficient of variation in respiratory intervals and the coefficient of variation in respiratory amplitudes from the time-series data of each respiratory curve.

#### 2.2.4. Determination of Appropriate CPAP Level

OSA is caused by obstruction or narrowing of the upper airway. When patients with OSA are treated with a sufficient CPAP level, the obstruction or narrowing of the upper airway is alleviated, leading to more regular respiratory movements. If the CPAP level is too high, patients may become uncomfortable and wake up. Therefore, to determine the appropriate CPAP level, we developed the RRI, which is a new index to evaluate the regularity of respiratory movements. The RRI quantifies the regularity of respiratory signals, and an increase in RRI is considered to indicate a greater complexity of respiratory patterns. When the RRI showed the lowest value, it was assumed that the upper airway obstruction had been sufficiently relieved. The CPAP level showing the lowest RRI was identified as the appropriate air pressure level for the patient in this study. After 2 months of appropriate CPAP therapy based on our daytime CPAP titration method utilizing the RRI, all patients with OSA were reexamined with night-time PSG using a CPAP device to evaluate the efficacy of the therapy.

### 2.3. Statistical Analysis

Results are presented as mean ± standard deviation (SD). The Wilcoxon matched-rank test was used to compare the values before and after CPAP therapy in patients with OSA (StatView, SAS Institute Inc., Cary, NC, USA). A value of *p* < 0.05 was considered to indicate statistical significance.

## 3. Results

### 3.1. Polysomnography of Daytime CPAP Titration

The mean TST during daytime CPAP titration was 108.2 ± 21.5 min. The distribution of sleep stages was 35.1 ± 18.2% (stage I), 51.0 ± 19.4% (stage II), 3.7 ± 3.6% (stages III and IV), and 10.3 ± 7.1% (REM). In 2 of 10 OSA patients, REM sleep could not be measured during daytime CPAP titration.

### 3.2. CPAP Level Determined by Daytime Titration Using the RRI

**Figure 1:**
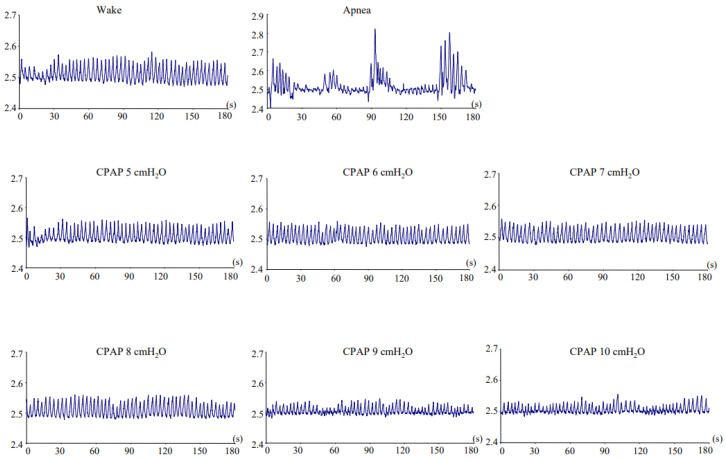
Respiratory movement curves for 3 min in a 67-year-old male patient with severe obstructive sleep apnea. This patient’s apnea–hypopnea index was 54.0 h^−1^. The vertical axis is shown in arbitrary units. CPAP, continuous positive airway pressure.

**Figure 2:**
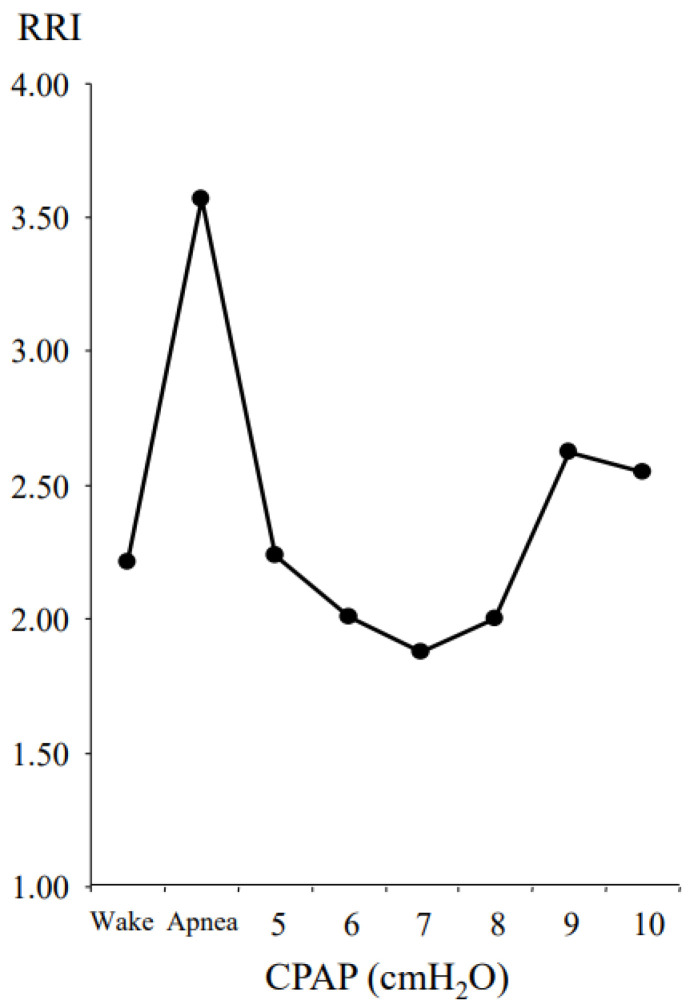
The RRI in a 67-year-old male patient with severe obstructive sleep apnea. The patient’s AHI was 54.0 h^−1^. The RRI indicated large values during apnea. We assumed that the appropriate CPAP level was determined as 7 cmH_2_O, which showed the lowest RRI. AHI, apnea–hypopnea index; CPAP, continuous positive airway pressure; RRI, respiratory regularity index.

Figure 1 shows the respiratory movement curves for 3 min in a 67-year-old male patient with severe OSA. Figure 2 shows the RRI in the condition of wake, apnea, and several CPAP levels. The RRI increased during apnea and decreased by using a CPAP device. Although the RRI was lowest at 7 cmH_2_O of CPAP, it again increased at 8, 9, and 10 cmH_2_O. This indicates that while CPAP alleviated the upper airway obstruction, excessive CPAP resulted in irregular respiratory movements. We assumed that the appropriate CPAP level was identified as the point where the RRI was lowest; in this case, the appropriate CPAP level was 7 cmH_2_O.

**Figure 3:**
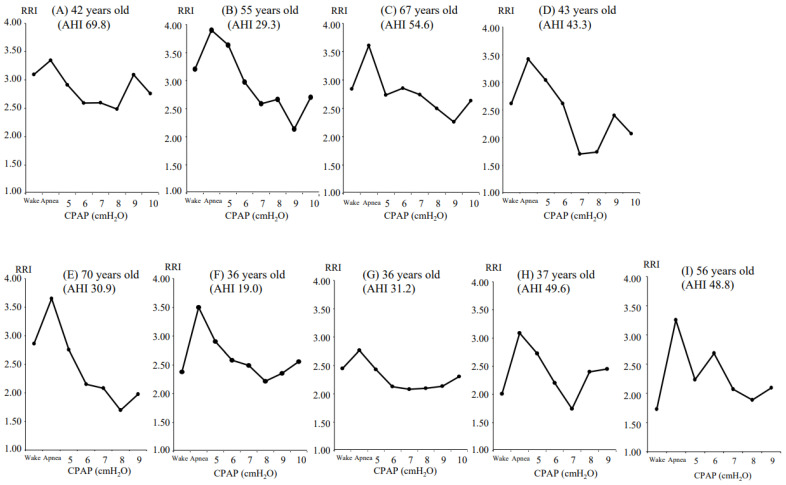
The RRI in each condition and the appropriate CPAP level identified for the remaining 9 patients. The appropriate pressure level of the CPAP device was identified as the level indicating the lowest RRI. The CPAP level with the lowest RRI was 8 (**A**), 9 (**B**), 9 (**C**), 7 (**D**), 8 (**E**), 8 (**F**), 7 (**G**), 7 (**H**), and 8 cmH_2_O (**I**), respectively. AHI, apnea–hypopnea index; CPAP, continuous positive airway pressure; RRI, respiratory regularity index.

Table 1 shows the values of RRI in each patient. Patients with OSA during sleep typically exhibit irregular respiratory efforts and RRI was higher, and treatment with an adequate CPAP level alleviates the airway obstruction or narrowing, which resulted in more regular respiratory patterns and RRI was lower. Figure 3 presents the RRI in each condition and the appropriate CPAP level identified for the remaining nine patients. We were able to determine the appropriate CPAP level in all study participants, with the CPAP level with the lowest RRI being 8 (A), 9 (B), 9 (C), 7 (D), 8 (E), 8 (F), 7 (G), 7 (H), and 8 cmH_2_O (I), respectively. The fixed air pressure level for the CPAP device was determined as 7.8 ± 0.7 cmH_2_O based on our daytime CPAP titration method using the RRI.

### 3.3. Efficacy of CPAP Therapy

We conducted another night-time PSG in study participants at the hospital after 2 months of the CPAP therapy determined based on the RRI. Table 2 shows the baseline characteristics of the patients and the efficacy results of CPAP therapy. The AHI (3.0 ± 2.1 h^−1^) during CPAP therapy was significantly improved compared to that before CPAP (43.1 ± 15.3 h^−1^) (*p* < 0.006). The arousal index was 44.2 ± 14.8 h^−1^ before CPAP, which decreased significantly during CPAP therapy (16.1 ± 7.8 h^−1^; *p* < 0.006). The percentages of sleep stage III and IV and REM sleep increased significantly during CPAP therapy. The Epworth Sleepiness Scale (ESS) scores [18] (12.6 ± 3.7) decreased significantly during CPAP therapy (4.2 ± 1.9; *p* < 0.005). The lowest SpO_2_ values in each sleep stage were also significantly improved by the fixed CPAP level based on our daytime CPAP titration method.

## 4. Discussion

OSA occurs when the upper airway becomes obstructed during sleep. Appropriate use of CPAP relieves upper airway obstruction and improves OSA. On the other hand, if CPAP levels are too high during sleep, the patient will wake up, and continuing with CPAP therapy becomes challenging. When there is upper airway obstruction during sleep and OSA, respiratory movements become labored and irregular, but once upper airway obstruction is removed, respiratory movements will become regular. The purpose of this study was to determine the appropriate CPAP level based on the RRI, which is a new index that quantifies the regularity of respiratory movement, and to evaluate the efficacy of the CPAP therapy utilizing this appropriate level. Our results demonstrated that the use of the CPAP level optimized with our daytime CPAP titration method, based on the CPAP level associated with the lowest RRI, was effective in patients with OSA. This approach led to a significant improvement of the AHI, and other indices, i.e., sleep stages, ESS, and lowest SpO_2_, also improved significantly.

Although CPAP therapy is widely used to treat OSA, sufficient therapeutic effects cannot be obtained if inappropriate settings are used. When CPAP levels are too high, issues with patient tolerance can occur. The current standard method for determining the air pressure level for CPAP devices is attended CPAP titration, in which an examiner performs PSG overnight and determines the CPAP level. This method involves gradually increasing air pressure by 1 cmH_2_O every 5 min or longer, starting with 4 cmH_2_O until snoring and hypopnea disappear. The final pressure obtained in this way is defined as the determined CPAP level [5]. A successful attended CPAP titration leads to observation of REM sleep and improvement of apnea. However, this method places a heavy burden on the examiner and may introduce subjectivity into the determination of the final CPAP level.

Alternative approaches have been reported, including split-night CPAP titration [6,7,8] and daytime CPAP titration [9,10,11]. In split-night CPAP titration, PSG is performed in the first half of the night, and CPAP titration is performed in the second half if OSA is suspected, reducing the hospital stay to only one day. MacArdle et al. have suggested that titration during REM sleep might not be necessary [6]. It is reported that daytime CPAP titration may be a useful alternative to conventional overnight titration for patients with OSA [9,10,11]. In this study, we conducted daytime CPAP titration, with a mean TST of 108.2 ± 21.5 min. Although REM sleep was not fully captured, effective CPAP levels were adequately determined using the RRI. In this study, the AHI improved significantly from before CPAP therapy to during CPAP therapy, along with ESS scores and lowest SpO_2_ values. These findings confirm the efficacy of our new method for CPAP titration.

Attended night-time CPAP titration places a heavy burden on the examiner, and there may be a risk of subjective interpretation, which results in deciding different CPAPs. Recently, automatic CPAP devices have been introduced clinically [19], and their usefulness in titration has also been reported [13,14]. Home telemedicine CPAP titration using an automatic CPAP device was reported to be as effective as standard care in achieving successful adaptation and patient compliance during the COVID-19 pandemic [20]. While automatic CPAP methods are convenient and effective, they can sometimes result in excessively high pressure levels, leading to patient discomfort. In contrast, our innovative RRI-based daytime CPAP titration method offers several advantages: it is cost-effective, requires significantly less time than conventional attended night-time CPAP titration, and maintains accuracy and convenience. Combining our method with automatic CPAP devices, e.g., setting the pressure range of automatic CPAP based on the appropriate CPAP level determined by our method, could further enhance the usefulness of this approach. On the other hand, since our method did not examine the RRI during REM sleep, it is necessary to conduct the study at night to evaluate RRI specifically during REM sleep.

## 5. Study Limitations

This study has several limitations, including the small number of participants and the fact that respiratory measurements were only taken during sleep stages I or II. A control group that received CPAP titration at night was not included. It is necessary to perform at night to assess the RRI during REM sleep because daytime CPAP titration cannot allow sufficient REM sleep for evaluation. Further studies will be needed to clarify the usefulness of our proposed objective approach to titrate CPAP levels in patients with OSA using regularity of respiratory movement as an indicator.

## 6. Conclusions

We introduced the RRI as a novel measure to quantify the regularity of respiratory movements. By applying this index, we could determine the effective CPAP level in patients with OSA. Our approach involved identifying the CPAP level that resulted in the lowest RRI during daytime CPAP titration. The findings show that using the fixed CPAP level associated with the lowest RRI leads to significant improvements in the AHI. In summary, the RRI-based daytime CPAP titration method used in our study can simplify the process of identifying the optimal CPAP level in patients with OSA.

## Figures and Tables

**Table 1:** The values of RRI in each patient with OSA.

				CPAP (cmH_2_O)					
Age	AHI (no./h)	Wake	Apnea	5	6	7	8	9	10
67	54.0	2.21	3.57	2.24	2.01	1.87	2.00	2.62	2.55
42	69.8	3.09	3.35	2.91	2.59	2.60	2.48	3.09	2.76
55	29.3	3.20	3.90	3.63	2.98	2.59	2.67	2.14	2.70
67	54.6	2.84	3.61	2.73	2.85	2.74	2.50	2.26	2.63
43	43.3	2.62	3.42	3.04	2.62	1.70	1.74	2.40	2.07
70	30.9	2.86	3.65	2.75	2.15	2.08	1.70	1.98	-
36	19.0	2.38	3.50	2.91	2.58	2.49	2.22	2.35	2.56
36	31.2	2.44	2.77	2.43	2.13	2.08	2.10	2.13	2.31
37	49.6	2.01	3.08	2.72	2.20	1.74	2.40	2.45	-
56	48.8	1.73	3.25	2.24	2.68	2.07	1.88	2.09	-

AHI, apnea–hypopnea index; CPAP, continuous positive airway pressure; OSA, obstructive sleep apnea; RRI, respiratory regularity index.

**Table 2:** Baseline characteristics of the patients with OSA and efficacy of CPAP.

	Before CPAP	During CPAP	*p* Value
AHI (no./h)	43.1 ± 15.3	3.0 ± 2.1	*p* < 0.006
Arousal index (no./h)	44.2 ± 14.8	16.1 ± 7.8	*p* < 0.006
ESS	12.6 ± 3.7	4.2 ± 1.9	*p* < 0.005
Total sleep time (min)	458 ± 82	444 ± 118	n.s.
Sleep stage (%)			
Stage I	34.8 ± 13.7	14.5 ± 6.1	*p* < 0.006
Stage II	44.9 ± 10.5	53.5 ± 6.7	*p* < 0.03
Stage III and IV	6.1 ± 4.3	11.6 ± 5.4	*p* < 0.02
REM sleep	14.2 ± 7.6	20.4 ± 9.9	*p* < 0.03
Lowest SpO_2_ during sleep (%)			
Stage I	84.1 ± 8.1	92.5 ± 1.7	*p* < 0.006
Stage II	83.7 ± 7.8	92.5 ± 1.7	*p* < 0.008
Stage III and IV	88.4 ± 7.6	94.4 ± 1.1	*p* < 0.005
REM sleep	81.1 ± 9.6	91.7 ± 2.4	*p* < 0.006

AHI, apnea–hypopnea index; CPAP, continuous positive airway pressure; ESS, Epworth Sleepiness Scale; n.s., not significant; OSA, obstructive sleep apnea; REM, rapid eye movement; SpO_2_, percutaneous arterial oxygen saturation. The Wilcoxon matched-rank test was used to compare the values before and during CPAP therapy.

## Data Availability

The data presented in this study are available from the corresponding author upon reasonable request.
